# Gene expression analysis at the onset of sex differentiation in turbot (*Scophthalmus maximus*)

**DOI:** 10.1186/s12864-015-2142-8

**Published:** 2015-11-18

**Authors:** Diego Robledo, Laia Ribas, Rosa Cal, Laura Sánchez, Francesc Piferrer, Paulino Martínez, Ana Viñas

**Affiliations:** Departamento de Genética, Facultad de Biología, Universidade de Santiago de Compostela, 15782 Santiago de Compostela, Spain; Institut de Ciències del Mar, Consejo Superior de Investigaciones Científicas (CSIC), 08003 Barcelona, Spain; Instituto Español de Oceanografía, Centro Oceanográfico de Vigo, 36390 Vigo, Spain; Departamento de Genética. Facultad de Veterinaria, Universidade de Santiago de Compostela, Campus de Lugo, 27002 Lugo, Spain

**Keywords:** Fish, Gonad, Development, *qPCR*, Genes, Sex ratio, Aromatase, Male, Female, Temperature

## Abstract

**Background:**

Controlling sex ratios is essential for the aquaculture industry, especially in those species with sex dimorphism for relevant productive traits, hence the importance of knowing how the sexual phenotype is established in fish. Turbot, a very important fish for the aquaculture industry in Europe, shows one of the largest sexual growth dimorphisms amongst marine cultured species, being all-female stocks a desirable goal for the industry. Although important knowledge has been achieved on the genetic basis of sex determination (SD) in this species, the master SD gene remains unknown and precise information on gene expression at the critical stage of sex differentiation is lacking. In the present work, we examined the expression profiles of 29 relevant genes related to sex differentiation, from 60 up to 135 days post fertilization (dpf), when gonads are differentiating. We also considered the influence of three temperature regimes on sex differentiation.

**Results:**

The first sex-related differences in molecular markers could be observed at 90 days post fertilization (dpf) and so we have called that time the onset of sex differentiation. Three genes were the first to show differential expression between males and females and also allowed us to sex turbot accurately at the onset of sex differentiation (90 dpf): *cyp19a1a, amh* and *vasa.* The expression of genes related to primordial germ cells (*vasa*, *gsdf*, *tdrd1*) started to increase between 75–90 dpf and *vasa* and *tdrd1* later presented higher expression in females (90-105 dpf). Two genes placed on the SD region of turbot (*sox2*, *fxr1*) did not show any expression pattern suggestive of a sex determining function. We also detected changes in the expression levels of several genes (*ctnnb1, cyp11a, dmrt2 or sox6*) depending on culture temperature.

**Conclusion:**

Our results enabled us to identify the first sex-associated genetic cues (*cyp19a1a, vasa* and *amh*) at the initial stages of gonad development in turbot (90 dpf) and to accurately sex turbot at this age, establishing the correspondence between gene expression profiles and histological sex. Furthermore, we profiled several genes involved in sex differentiation and found specific temperature effects on their expression.

**Electronic supplementary material:**

The online version of this article (doi:10.1186/s12864-015-2142-8) contains supplementary material, which is available to authorized users.

## Background

Sex was thought to have arisen in a single evolutive event in the last common ancestor of all eukaryotes, since sexual reproduction is almost universal and exclusive of this group [1]. Considering its consequences over the lifespan of an organism and its influence on population demography, it is thought that the sex determination (SD) mechanism should be under strong selection forces [2]. However, sex can be established by many different and fast-evolving mechanisms [3, 4], indicating that SD triggers have emerged several times throughout evolution [5]. Within vertebrates, different sex determining systems have been described. In therian mammals, with a XX/XY chromosome system, sex depends on the presence of the *Sry* gene, a paralogue of *SOX3,* on the Y chromosome [6], while in birds (chicken) with a ZZ/ZW chromosome system, the *DMRT1* gene with a double dosage is required for testis development [7]. Also, in *Xenopus laevis* the *DM-W* gene, a paralogue of *DMRT1*, is responsible for SD [8]. When *dmY,* belonging to the DM family of transcription factors like *DMRT1* and *DM-W*, was found to be the sex determining gene (SDG) of the fish *Oryzias latipes*, a biased and recurrent recruitment of specific SDGs or families throughout evolution was suggested [9]. However, later findings in fish do not seem to support this hypothesis.

Fish, with around 30,000 species [10], is the most diverse group of vertebrates and its study has broadened our knowledge on SD. Fish diversity is also reflected by the variety of reproductive strategies: unisexuality, different types of hermaphroditism and gonochorism; and also by the diversity of SD mechanisms [11]. In the last years, an important effort has been made in order to identify the SDG in several model and aquaculture fish species. Different productive traits are sex-associated in farm fish such as growth rate, color, taste and flesh quality; hence, the interest of the industry in producing monosex stocks [12]. Detailed information at gene level is available for only a limited number of fish species. Five different master SDGs have been identified so far*: dmY /dmrt1by* in *Oryzias latipes* and in *O. curvinotus* [13], *gsdf* in *O. luzonensis* [14], *amhy* in *Odontesthes hatchery* [15], *amhr2* in *Takifugu rubripes*, *T. pardalis* and *T. poecilonotus* [16], and *sdY* in salmonid family [17]. Recently, a distant cis-regulatory element of *Sox3* necessary for male determination in *O. dancena*, a species with a XX/XY SD system, has also been identified [18], and *dmrt1* has been suggested as the SDG in *Cynoglossus semilaevis* [19]. However, little information is available, not only on the SDG, but also on the initial molecular pathways related to sexual differentiation.

Traditionally, SD has been related to the switching mechanism of a hierarchical genetic network that causes the activation of downstream genes involved in gonad differentiation (GD) leading to the differentiation of testes or ovaries [20]. Thus, concerning whether the first difference between sexes is the expression of a gene/s or the strength of an environmental factor, SD can be genetic or environmental, although both ways can coexist [12, 21]. In the classical view of SD and GD, the downstream genes of the cascade were assumed to be highly conserved, and only the genes at the top of the cascade would change by gene duplication (and by the recruitment of a downstream gene) or by allelic diversification, establishing a new SD mechanism [20]. Nowadays, the conservation of the downstream cascade has been questioned [22, 23] and a new view, which considers sex as a threshold phenotype in which both genetic and environmental factors can act alone or in combination and, importantly, at different times during the period of GD is gaining support [4, 24]. In this new view, SD encompasses not only the initial trigger, be it genetic, environmental or both, but also the whole GD process, and different factors such as cell proliferation and hormone levels would be involved in determining a threshold which would give rise to a testis or an ovary, thus fitting to a threshold quantitative trait [4, 12].

Turbot is one of the most important species cultured in Europe, being Galicia (North-West of Spain) the main production region since the eighties. Production and quality of farmed fish rely on a deep knowledge of biological functions, especially those related to reproduction, growth and disease resistance. In this context, the production of monosex stocks to exploit sex-associated dimorphisms related to productive traits, especially growth and sexual maturation, has been a sought in finfish aquaculture by different approaches [11, 25]. Turbot shows one of the strongest sexual growth dimorphisms amongst marine species and females can reach up to 50 % bigger size than males [26], thus industry is interested in the production of all-female populations. In the last years, an important effort has been devoted to understanding SD and GD in this species. Analysis on mitotic and meiotic chromosomes revealed the absence of an heteromorphic sex chromosome pair related to sex [27, 28]. The major SD region was located on the linkage group (LG) 5 at 2.6 cM of Sma-USC30 marker (R^2^ = 86.1 %) [29], but other minor sex-related quantitative trait locus (QTLs) were detected at LG6, LG8 and LG21 [30]. In that study, a ZZ/ZW chromosome system was established in accordance with the sex ratios of progenies obtained from hormonally sex-reversed parents [31]. Temperature also showed a minor influence on sex ratios in this species [31]. Close to the sex-associated marker Sma-USC30 several candidate genes were identified (*sox2*, *dnajc19*, *fxr1*, *atp11b*, *fkbp2* and *dlg1*), but eventually discarded because no association to sex was detected at the species level, so the SD gene remains unidentified in turbot [32]. Considering the lack of information on the SD mechanism of turbot, we determined the expression patterns of a suite of 29 genes shown to be involved in GD in other species at the initial critical stages of sex differentiation using a large amount of fish and sampling times in turbot. In a previous study, we analyzed reproduction in this species through the use of oligo-microarrays, spanning a larger age period but in a lower number of samples and finding several genes involved in ovary or testis development (Ribas et al. submitted). In this work, our aim was to study gene expression at a very specific time point coinciding with the onset of sex differentiation. We also evaluated the effect of temperature to clarify whether it has a major role in turbot SD and its possible interaction with genetic factors. Our results enabled us to establish the correspondence between gene expression profiles and histological sex and to identify the first sex-associated genetic cues at the initial stages of gonad development in turbot.

## Results

### Sampling and sexing

**Fig. 1:**
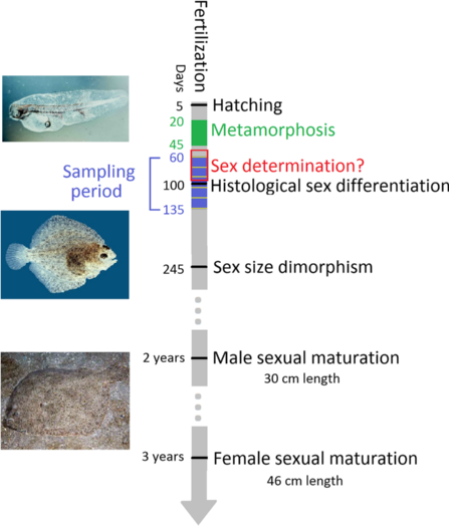
Turbot gonad differentiation. Main histological and physiological events along sexual differentiation in turbot. Turbot images were obtained from http://larvalbase.org/and http://fishbase.se/

A timeline of turbot gonad differentiation is shown in Fig. 1. All of the 180 turbot samples used in this study were genetically sexed using the Sma-USC30 marker and, additionally, the 105, 120 and 135 dpf samples were histologically sexed. Eighty-nine females and eighty-five males could be genetically sexed because the Sma-USC30 marker was informative, the remaining six samples being removed from this analysis since they could not be sexed. A 7 % sexing discrepancy was observed between the genetic and histological information in the samples obtained at 105, 120 and 135 dpf. Sma-USC30 is expected to present a 2 % sex-genotyping error [30], so in our data the 5 % sex allocation error may represent sex-reversal events due the action of a secondary QTL or environmental factors. However, given the reasonable accuracy of genetic sexing, the sex of samples below 105 dpf obtained through SmaUSC-E30 genotyping was considered for further analyses.

Males and females did not show length differences both in the whole dataset and at each age (Mann–Whitney test, *P* < 0.05) (Additional file 1). However, significant differences were found at every age between the three temperatures, except at 90 and 120 dpf, where 18 °C-reared animals did not differ in length from those reared at 23 °C (Table 1).

**Table 1:** Length comparison between temperatures for each age group

Age (dpf)	Temperature (°C)	Mean length ± SD (cm)	Percentage (%) / 18 °C	*P* value/18 °C
60	15	3.14 ± 0.27	91.2	0.020
	18	3.44 ± 0.26	100	-
	23	3.72 ± 0.18	108.8	0.013
75	15	3.63 ± 0.43	87.8	0.044
	18	4.13 ± 0.45	100	-
	23	4.73 ± 0.38	114.6	0.006
90	15	4.95 ± 0.44	83.05	0.001
	18	5.90 ± 0.42	100	-
	23	6.02 ± 0.52	101.7	0.622
105	15	7.21 ± 0.68	73.5	0.000
	18	9.76 ± 0.51	100	-
	23	10.75 ± 0.47	109.2	0.000
120	15	8.33 ± 0.37	73.5	0.000
	18	11.26 ± 0.70	100	-
	23	11.85 ± 0.68	104.4	0.103
135	15	9.57 ± 0.33	78.1	0.000
	18	12.34 ± 0.36	100	-
	23	13.28 ± 0.71	108.1	0.003

Table 1: mean length in cm and standard deviation (SD) for each turbot stage-temperature group. The percentage length difference for 15 and 23 °C groups referenced to the 18 °C group and the *p* value of 15 and 23 °C temperature lengths compared to18 °C are also shown

#### Co-localization of targeted genes with sex-related QTLs

**Fig. 2:**
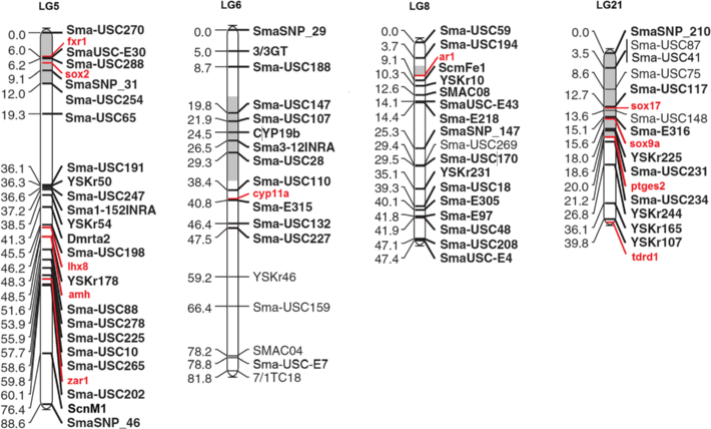
Turbot sex QTLs and target genes. Four turbot linkage groups are shown. Estimated location of the target genes is shown in red. Grey shaded LG areas represent the position of the SD QTLs [29, 30]

A main SD QTL in linkage group 5 (LG 5) and three minor ones in LG6, LG8 and LG21 were previously reported in turbot [29, 30]. After establishing the relationship between the turbot map (linkage groups) and the turbot genome (scaffolds; Figueras et al., unpublished), 11 genes in the subset analyzed here (Additional file 2 for genes and gene functions) could be located in LGs harboring a SD QTL (Fig. 2). Five genes were found in LG5 and two of them, *sox2* and *fxr1*, co-localized with the main SD QTL; *ar1* co-localized with the sex QTL in LG8; four genes were placed in LG21 and two of them, *sox9a* and *sox17*, within the confidence interval of the SD QTL.

### Global expression patterns

**Fig. 3:**
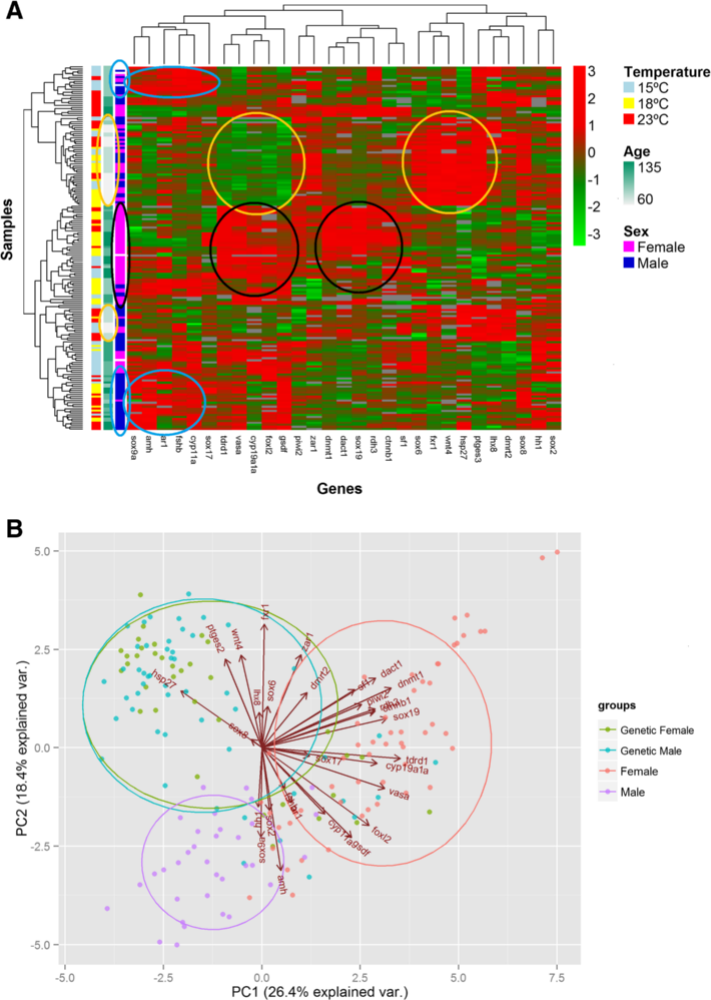
Global expression patterns. **a** Heatmap of target genes and all gonad samples. Gene names are shown in the bottom of the figure while gene hierarchical cluster is shown in the top. Log fold change expression values representation ranges from red (highest expression) to light green (lowest expression). Sample names are not shown, instead each sample is represented by the three colors at the left of the figure which indicate sex (magenta for females, and blue for males), age (ranging from 60 to 135 dpf corresponding to a scale going from grey to dark green) and temperature (light blue for 15 °C, yellow for 18 °C and red for 23 °C). Yellow, black or blue circles highlight expression patterns characteristic of undifferentiated, female or male individuals respectively. **b** Samples were grouped according to the fold change expression values of the target genes by a principal component analysis. Samples labeled as “Female” and “Male” and colored in red and purple, respectively, represent gonad samples which were both genetically and histologically sexed because developing testis and ovaries could be distinguished from each other. Samples labeled as “GenFemale” and “GenMale” and colored in olive green and light blue, respectively, are gonad samples which were only genetically sexed because histologically the gonads were still undifferentiated. A 66 % of the samples of each group are expected to be placed in their respective circles. The arrows with the name of the genes at the end represent how each gene contributes to the two principal analysis components represented in the figure

Samples and genes were hierarchically clustered in a heatmap using the Pearson correlation coefficient as distance measure (Fig. 3a). For each sample, rearing temperature, age and sex are shown in the heatmap. Some samples are grouped according to sex or age and can be associated with particular groups of coexpressed genes. The samples of 60 and 75 dpf are clustered in two groups (labelled in grey, within two yellow circles) and they are characterized by the high expression of *sox6*, *fxr1*, *wnt4*, *hsp27*, *ptges3, T4_30483 and dmrt2* (yellow circles on the right), but also by the nearly null expression of *tdrd1*, *vasa*, *cyp19a1a*, *foxl2* and *gsdf*, involved in gonad maturation and female differentiation (yellow circle on the left). These samples are grouped by age independently of temperature or sex, which do not seem to represent relevant factors on the diagnostic genes expressed at these ages. Two different groups of older fish, one essentially made of females and another essentially made of males, could clearly be identified. The female group (black circle on the right) is mainly associated with the up-regulation of two different clusters of genes: one cluster containing *cyp19a1a*, *foxl2*, *vasa*, *tdrd1* and *gsdf*, genes not expressed in undifferentiated individuals, as outlined before; and another cluster containing *dnmt1*, *dact1*, *sox19*, *rdh3* and *ctnnb1* (black circle on the left). The male group (blue circles) is associated with the expression of *sox9*, *amh*, *ar1*, *fshb, cyp11a* (blue circle at the bottom). These “male” genes are also highly expressed in a mix of males and females of around 90 dpf and mostly reared at low temperatures (blue circle at the top). Some “female” genes (*foxl2*, *gsdf*, *vasa*, *tdrd1*) are also expressed to a lower extent in the male samples, suggesting a role in gonad development irrespective of sex (next to the blue circle at the bottom). As previously mentioned, some blocks connected to rearing temperature can also be seen, but, in general, it does not seem to be a determining factor for sample clustering.

PCA analysis on the 180 samples (Fig. 3b) revealed that histologically sexed males and females (≥105 dpf; referred to as males and females in the figure), could be in most cases clearly discriminated by their differential expression. However, younger individuals (60–90 dpf; only genetically sexed and referred to as genetic males and females in the figure), appeared fully overlapped in the PCA, likely because they are still undifferentiated. A 66 % prediction ellipse for each group is shown in Fig. 3b indicating that if new individuals were added to our analysis from a certain group, 66 % of them would expect to be placed inside the corresponding ellipse. Some of the genetically sexed individuals (60, 75 and 90 dpf) are also found in the ellipses drawn for phenotypic males or females which, also considering the previous heatmap results, indicates that turbot GD might start before 105 dpf. Interestingly, about half a dozen genetic males were included in the female circle. The arrows indicate the weight of each gene on the two first principal components. Among the analyzed genes there is a large group seemingly contributing to female differentiation (e.g., *cyp19a1a*, *sox19*, *tdrd1*, *dact1*), while the presumed male-related genes are fewer and not so markedly pointing towards male differentiation (*sox9*, *amh*, *sox2*, *hh1*). Also, as suggested in the heatmap, some genes are clearly related to undifferentiated individuals (*sox6*, *fxr1*, *wnt4*, *hsp27*, *ptges*).

### Sex differences

**Fig. 4:**
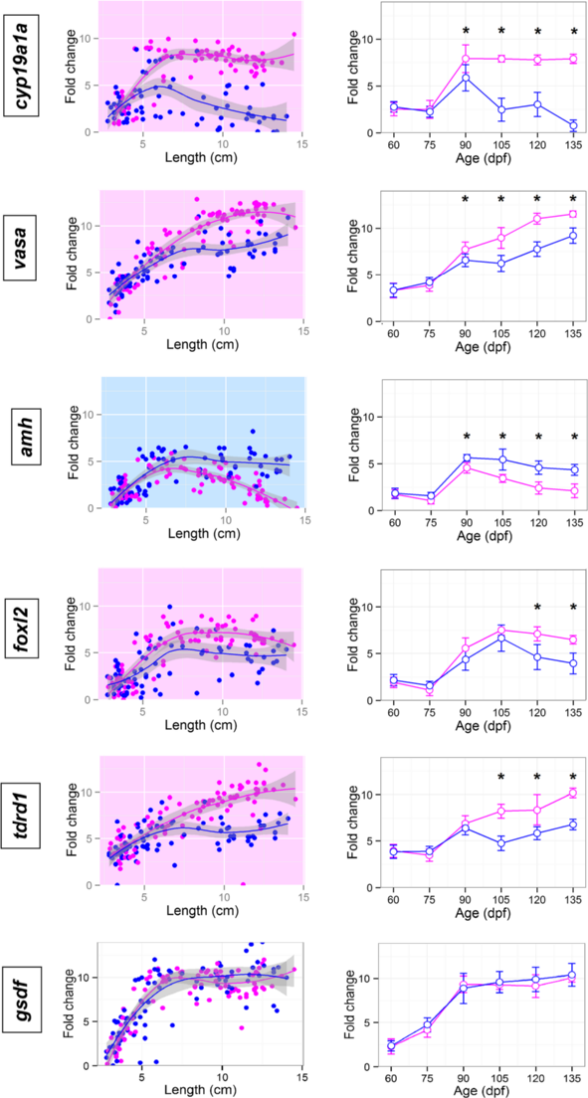
Gene fold change values along gonad development. *Cyp19a1a*, *vasa*, *amh*, *foxl2*, *tdrd1* and *gsdf* fold change values for each sample plotted according to both its length, in cm, and its age, in days post fertilization. Female samples are shown in magenta and male samples in blue. In the FC/length figure for each gene non-linear trend lines were calculated by loess regression and genes with significative differences between sexes at any age point present a pink background if the gene is up-regulated in females or a blue one if it is up-regulated in males. Genes without sex differences have a white background. In the FC/age figure, error bars represent the standard error of the mean, also an asterisk marks those age points were the differences in expression between males and females are significant

**Fig. 5:**
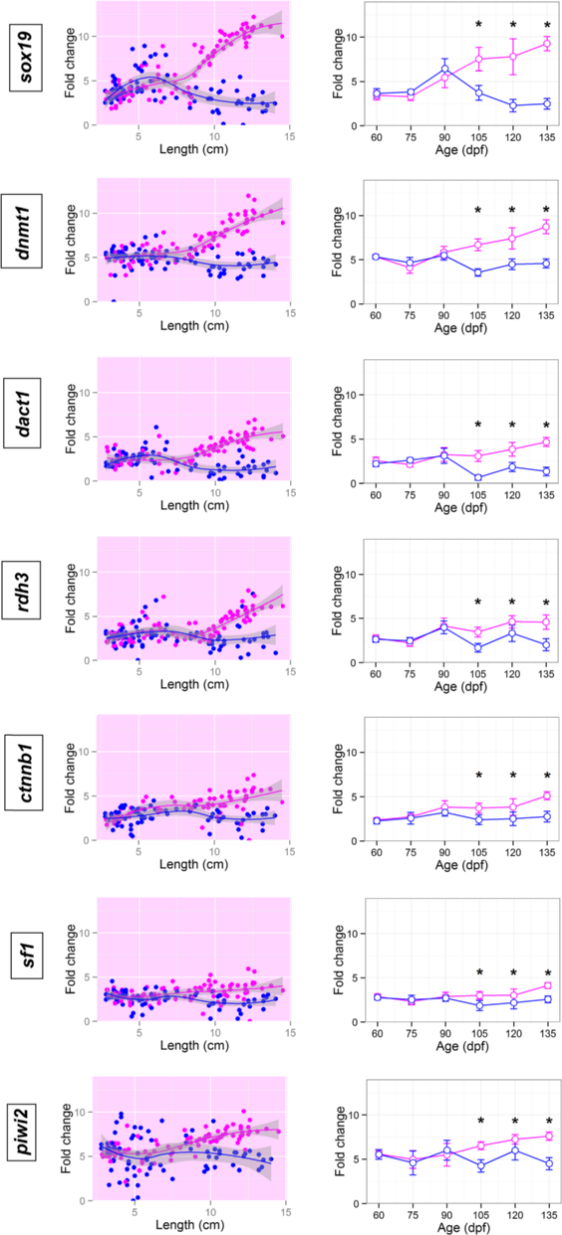
Gene fold change values along gonad development. *Sox19*, *dnmt1*, *dact1*, *rdh3*, *ctnnb1*, *sf1* and *piwi2* fold change values for each sample plotted according to both its length, in cm, and its age, in days post fertilization. Female samples are shown in magenta and male samples in blue. In the FC/length figure for each gene non-linear trend lines were calculated by loess regression and genes with significative differences between sexes at any age point present a pink background if the gene is up-regulated in females or a blue one if it is up-regulated in males. Genes without sex differences have a white background. In the FC/age figure, error bars represent the standard error of the mean, also an asterisk marks those age points were the differences in expression between males and females are significant

**Fig. 6:**
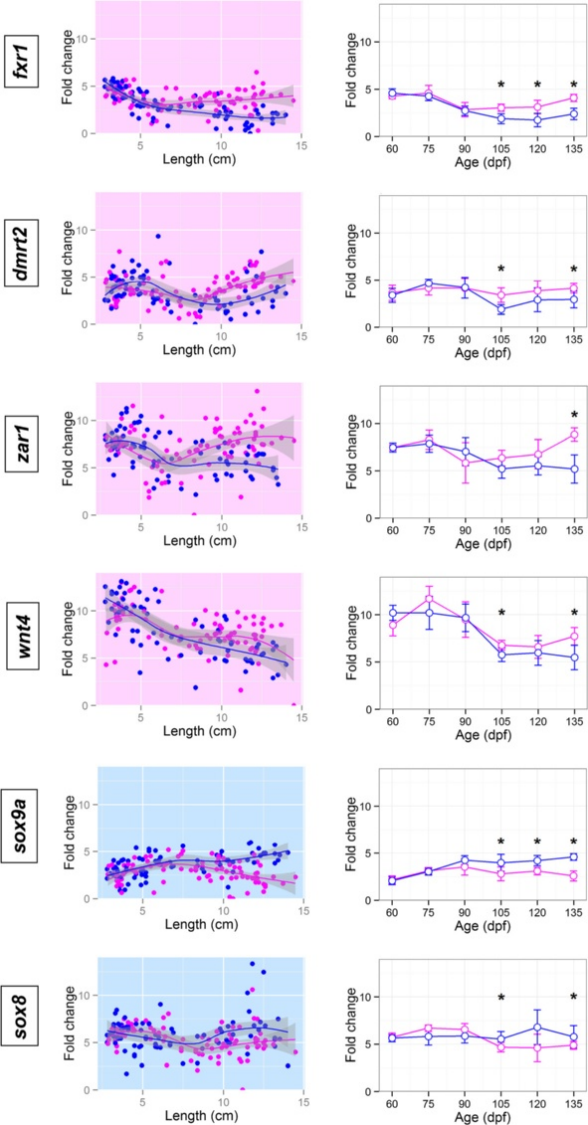
Gene fold change values along gonad development. *Fxr1*, *dmrt2*, *zar1*, *wnt4*, *sox9a*, *sox8* fold change values for each sample plotted according to both its length, in cm, and its age, in days post fertilization. Female samples are shown in magenta and male samples in blue. In the FC/length figure for each gene non-linear trend lines were calculated by loess regression and genes with significative differences between sexes at any age point present a pink background if the gene is up-regulated in females or a blue one if it is up-regulated in males. Genes without sex differences have a white background. In the FC/age figure, error bars represent the standard error of the mean, also an asterisk marks those age points were the differences in expression between males and females are significant

Fold change (FC) expression values for those differentially expressed genes between males and females (genetic sex for 60 to 90 dpf samples, phenotypic sex for 105 to 135 dpf) were analyzed in relation to age (Mann–Whitney test; *P* < 0.05) (Figs. 4, 5 and 6).

Sex differences were first observed at 90 dpf, when *cyp19a1a* (FC_F/M_ = 2.0) and *vasa* (FC_F/M_ = 1.2) (Fig. 4) which were more expressed in females, and *amh* (FC_M/F_ = 1.1) in males (Fig. 4). These three genes also increased their expression from 75 to 90 dpf, in both sexes (~5 cm), which is also observed for *foxl2*, *tdrd1*, *gsdf* (Fig. 4) and *sox19* and *rdh3* (Fig. 5). *Gsdf* expression increased dramatically even earlier, from 60 to 75 dpf (FC_75/60_ = 4.3, ~2.5 cm). *Foxl2* did not show significant differences in expression in females until 120 dpf, mainly because its expression decreased slowly in males (Fig. 4). *Tdrd1* pattern of expression resembled that of *vasa*, being more highly expressed in females at 105 dpf and onwards. Other genes increased their expression at 105 dpf (~9 cm length) in females, while their expression remained low or even decreased in males. Fold change values of females vs. males at 105 dpf were above 1 for *sox19* (FC_F/M_ = 3.8), *dnmt1* (FC_F/M_ = 3.1), *dact1* (FC_F/M_ = 2.4), *rdh3* (FC_F/M_ = 1.8), *ctnnb1* (FC_F/M_ = 1.3), *sf1* (FC_F/M_ = 1.1) and *piwi2* (FC_F/M_ = 2.3) (Fig. 5) and these differences increased from 105 to 135 dpf, when the FCs were between 2 and 7 for the seven genes. *Fxr1* was also up-regulated in females at 105 dpf (FC_F/M_ = 1.2) and showed a 47 % increase at 135 dpf (Fig. 6), but presented higher expression levels in undifferentiated individuals, irrespective of sex. A similar pattern was also observed for *wnt4, dmrt2 and zar1* (Fig. 6), genes which at some point during sex differentiation, 105–135 dpf (above 10 cm length), showed a higher expression in females, but its expression decreased from 75 to 90 dpf (~5 cm, Fig. 6).

In contrast to that observed in females, in our study there were very few genes in the subset assayed whose expression were higher in males and, even in these cases, the differences between males and females were low. One of them was *sox9a* for which sex differences increased from 105 dpf (FC_M/F_ = 1.2, ~11 cm) up to 135 dpf (FC_M/F_ = 2.0) (Fig. 6). Another *sox* family gene, *sox8*, highly expressed in undifferentiated individuals in both sexes, was up-regulated in males at 105 dpf (FC_M/F_ = 0.8), but the difference remained constant at 135 dpf (Fig. 6). Two additional genes, *fshb* and *cyp11a*, showed mean expression values slightly higher in males, but not significant (Additional file 3).

Some genes like *ptges3, hh1, hsp27* or *T4_30483* did not show sex differences (Additional file 3). Among these, a gene of the *sox* family, *sox17*, showed some groups of outliers whose expression was not explained either by sex or by length/age. Other two genes of this family, *sox2* and *sox6*, did not present any clear expression pattern along development or by sex, and androgen receptor 1, *ar1* did not show sex dimorphic expression either.

### Discriminant analysis

**Fig. 7:**
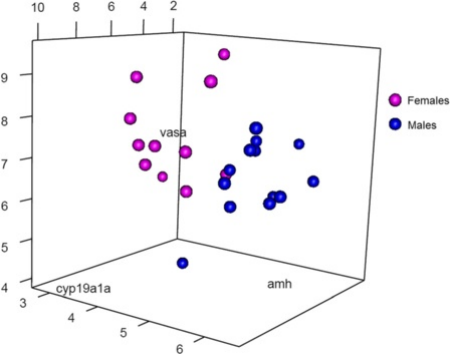
Discriminant analysis 3D plot. Ninety days post fertilization samples were plotted in a three dimensions graph according to their fold change values for *cyp19a1a*, *amh* and *vasa*. Female samples are colored in magenta and males in blue

A discriminant analysis considering the earliest dimorphic expressed genes -*cyp19a1a*, *amh* and *vasa*– correctly classified 100 % of the genetic males and 82 % of the genetic females at 90 dpf, representing as a whole 91 % of individuals correctly classified (Fig. 7). The remaining 18 % of the genetic females were grouped as males. These three genes can constitute a fairly efficient genetic tool for early sexing of turbot. Furthermore, from 105 dpf onwards (sexed by histology), the expression of *cyp19a1a* alone is capable of perfectly discriminating males and females without error.

### Network analysis

**Fig. 8:**
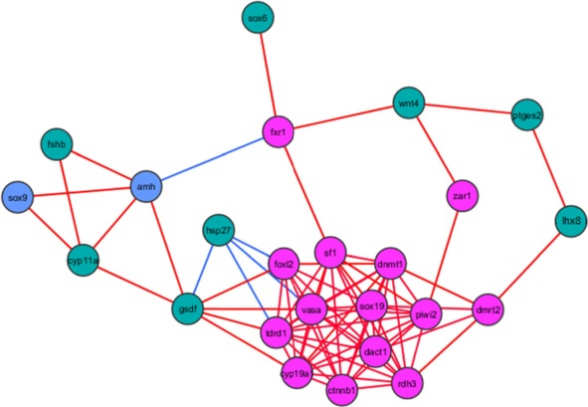
Network representation. Weighed correlation network performed with the fold change expression values of the genes is shown. Genes are represented as blue circles if they are up-regulated in males at any age, magenta if they are up-regulated in females, or dark green if no differences were found. Lines connecting genes indicate significant correlations, red lines are positive correlations and blue lines are negative correlations

To further understand the relationships between genes we performed a network analysis based on gene-to-gene correlations (Fig. 8). A tight cluster with several female up-regulated genes (e.g., *cyp19a1a*, *foxl2*, *vasa*, *sox19*, *ctnnb1*) was found with all their genes inter-connected. Also, *sox9* and *amh* constituted a small male-like cluster together with *fshb* and *cyp11a*. The two clusters were connected through two genes: *fxr1*, gene located at the main sex determining region of turbot, and *gsdf*. The absence of some genes in the network (*sox2*, *sox8*, *sox17*, *ar1*) suggests that they did not show significant relationships with any other gene in our analysis and for the chosen correlation threshold. This does not mean that they do not have any role in sex differentiation, since our study analyzed the expression of a limited number of genes (29). If more genes were added, it is possible that these genes could have shown connection to the network through them.

### Temperature effects on gene expression

**Fig. 9:**
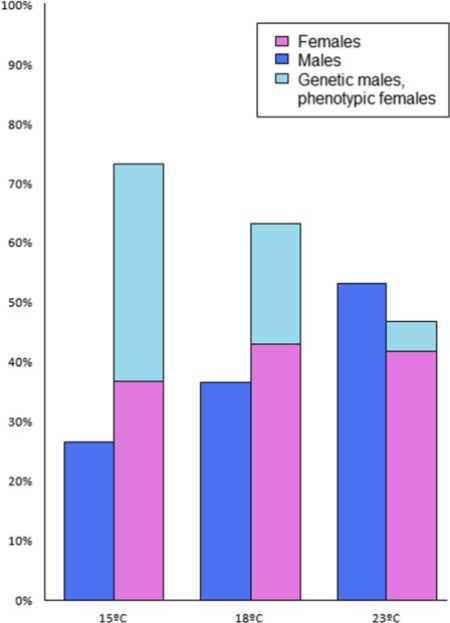
Sex proportions and temperature. Percentage of male and female turbot, histologically sexed, at 15, 18 and 23 °C. Also, the percentage of phenotypic females which are genetic males is shown. No genetic females developed as males. Sample size = 30 fish per temperature

**Fig. 10:**
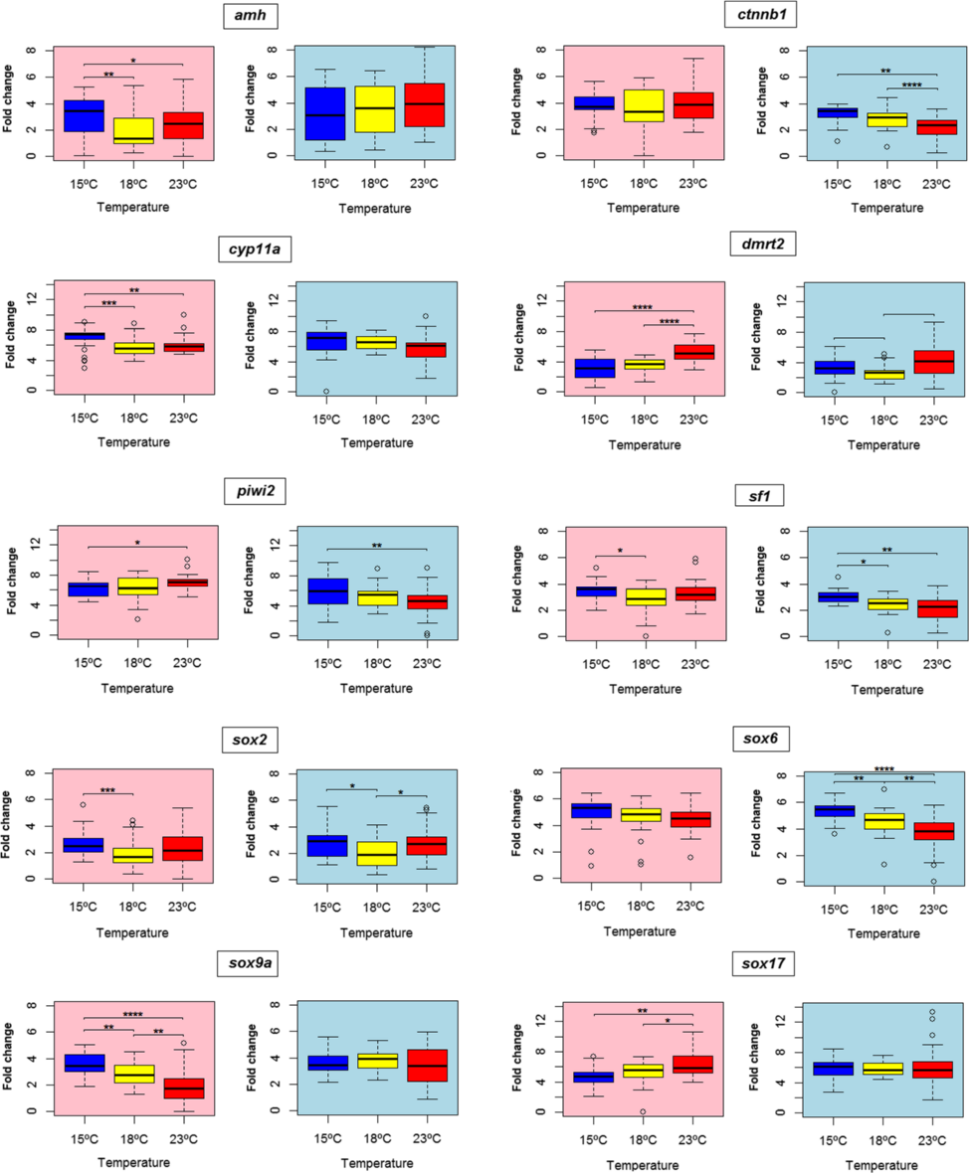
Temperature effects on gene expression. Mean fold change gene expression values at 15, 18 and 23 °C in the whole dataset are shown for males (light blue background) and females (pink background). Error bars represent standard deviation. Significate differences between temperatures are indicated by *(0.01 < *p* < 0.05), **(0.001 < *p* < 0.01), ***(0.0001 < *p* < 0.001) or ****(*p* < 0.0001). Black asterisks indicate that the detected difference is independent of fish length, while red asterisks indicate that fish length has an influence on the temperature differences

We found a higher proportion of females at both 15 °C and 18 °C than at 23 °C, where the male:female proportion was close to 1:1 (Fig. 9). However, these differences were not significant (Chi-square test, *p* = 0.11). Genotyping of the SD marker in the main SD QTL in LG5 strongly suggested that some genetic males developed as females: a total of 16 genetic males were classified as females by histology. This discrepancy can either be caused by 1) temperature effects on SD or 2) interaction of a secondary QTL (located on LG6, LG8 and LG21) with the main SD QTL in LG5. Further, we analyzed the effects of temperature on gene expression in males and females separately and, since turbot length was different between temperatures in almost every development stage, we checked if the detected temperature differences in gene expression were independent of length (Fig. 10) or not (Additional file 4). Among those genes with length-independent temperature effects on expression (Fig. 10), only *sox2* showed temperature effects which are not sex dependent. This gene showed higher expression at 15 and 23 °C, although the difference between 18 and 23 °C was not significant in females. Among those genes showing sex-specific temperature effects *amh*, *sox9a* and *cyp11a* were more highly expressed at low temperatures in females, while *sox17* and *dmrt2* showed the opposite pattern with higher expression at 23 °C in females. On the contrary, *ctnnb1*, *piwi2*, *sf1* and *sox6* were up-regulated at low temperatures in males, and the four genes showed a very similar pattern.

Finally, since 90 dpf is the first age where we find sex dimorphic expression and can be considered the onset of sex differentiation, we decided to test for temperature differences at this age which were independent of both length and sex, since most genes did not show dimorphic gene expression at this age. A single gene showed significant differences between temperatures at 90 dpf: *ctnnb1* (Additional file 5). *Ctnnb1* highest expression was observed at 18 °C, while its lowest expression was at 23 °C.

## Discussion

Recently, sex determination and differentiation have begun to be perceived as a single modular process rather than a cascade, where sex behaves in many instances as a threshold-like character [33, 34]. Under this view, gonad fate depends on several factors acting coordinately, including many genes and possibly also environmental variables. In this sense, and given the interest in obtaining monosex stocks in many finfish aquaculture species, understanding the different gene patterns during early sex differentiation, supposedly the moment where gonad fate can be more easily controlled or altered, is of great importance to manipulate sex determination in order to control sex ratios. In this study we have analyzed the expression of 29 genes previously connected to sex differentiation in other species, some of them studied in fish regarding sex differentiation for the first time. A total of 21 genes were found to show dimorphic expression at some point during early sex differentiation in turbot. The influence of temperature was also assessed, finding differences between temperatures for 10 genes. This study has broadened our knowledge of gene expression patterns during early sex determination in turbot in particular and in fish in general.

### Early sex differentiation and primodial germ cells

Although morphological gonad differences between sexes were not detected before 105 dpf, the first molecular signs of sex differentiation were observed between 75 and 90 dpf (5–6 cm length), characterized by an expression increase of *gsdf* and *tdrd1*, but also by the expression increase and differential expression of *cyp19a1a*, *amh* and *vasa*, which allowed discriminating males (high *amh* expression) and females (high *cyp19a1a* and *vasa* expression) at 90 dpf. *Vasa*, *tdrd1* and *gsdf* are genes related to primordial germ cell development. *Tdrd1* proteins were detected in the primordial germ cells of zebrafish (*Danio rerio*) at 4 dpf and were involved in both oocyte and sperm development [35]. T*drd1* was found to interact with *vasa,* which is also a highly specific marker of germ cells [36–40] required for their development [41] and conserved along several invertebrate and vertebrate taxa [42]. While *vasa* and *tdrd1* are specific germ cell markers, *gsdf* is a teleost-specific member of the TGF-β superfamily which has been reported to be expressed in the somatic cells surrounding the primordial germ cells in rainbow trout and promoting their proliferation [43]. *Gsdf* has shown higher levels of expression in testis in zebrafish, three-spot wrasse (*Halichoeres trimaculatus*) and coelacanth (*Latimeria menadoensis*) [44–46]. Furthermore, *gsdf* has been proposed as the male sex determining gene in *Anoploma fimbria* [47], and a copy named *gsdf*^*Y*^ has been found to be the sex determinant gene in *Oryzias luzonensis,* with a higher expression in males 10 days after hatching [14]. *Gsdf* has also been found to be directly up-regulated by *dmy*, male SDG of *Oryzias latipes* [48], and *sox3*, male SDG of *Oryzias dancena* [18] during the first stages of GD. Although *gsdf* does not seem to have such a male-like function in turbot, since it did not present sex dimorphic expression, it seems to be important for gonad development in both sexes since its expression greatly increased between 75 and 90 dpf. Even more, this is the only gene which showed a significant expression increase from 60 to 75 dpf in our study, consistent with a function as germ cell inductor since its expression increase precedes that of *vasa* or *tdrd1*.

The expression patterns of *vasa*, *tdrd1* and *gsdf* in turbot suggested that primordial germ cells start proliferating between 75 and 90 dpf in both sexes, either faster in females or suffering a certain delay in males, as suggested by *vasa* and *tdrd1* expression levels. Another germ-line specific gene, *piwi2*, was investigated in our study, but it showed a different expression pattern and its induction was limited to females and delayed until 105 dpf. In zebrafish, *piwi2* has been found to play a crucial role in meiosis [49] and perhaps its different pattern of expression in turbot may be related to the onset of meiosis in female germ cells.

The amount of primordial cells is recognized as one of the initial differences between male and female gonads in some fish species like zebrafish [50], medaka [51] and stickleback (*Gasterosteus aculeatus*) [52], although not in others such as loach (*Misgurnus anguillicaudatus*) [53] and goldfish (*Carassius auratus*) [54]. Further, germ cell proliferation has been found to be associated with SD in several fish species. In medaka, germ cell proliferation is inhibited in males when the sex determining gene, *dmY*, is expressed at the SD stage before testis differentiation [23]. When *dmy* is not active in XY embryos, germ cells proliferate and enter meiosis as in XX embryos. Surprisingly, *amh* does not present a sex dimorphic expression in medaka during GD [55]. Although the Müllerian ducts are not present in modern teleosts [56], *amh* orthologs have been described in fish and characterized as gonad specific key factors for male sex differentiation [55, 57, 58]. Yet, even if *amh* does not present sex dimorphic expression in medaka, it has been found to control germ cell proliferation in this species, and mutations on its receptor (*amhrII*) lead to excessive proliferation of germ cells which caused male-to-female sex reversal [59], although phenotypic female XY gonads still expressed *dmY*. Furthermore, if these *amhrII* mutants are depleted of germ cells, testis development takes place [60]. Thus, *amh* seems to be a repressor of germ cell proliferation in medaka and to play a major role in sex determination. This is also the case in fugu (*Fugu rubripes*), where a single SNP in the coding region of *amhrII* is likely responsible for SD [16]. This SNP encodes a protein with a reduced function and is fixed in females, which are not sensitive to *amh* [16]. Also, in the Patagonian pejerrey, a copy of *amh*, *amhY*, has been found to be the sex determining gene and its action has been suggested to regulate germ cell proliferation, being upstream to the autosomal *amh* and relegating the former to a function in testicular maturation and/or spermatogenesis [15].

Interestingly, in the female gonad of zebrafish the maintenance of *cyp19a1a* expression, but not its activation, has been related to the presence of the primordial germ cells [50], pointing towards a model where *amh* is responsible for the control of germ cell proliferation while germ cells aid to maintain *cyp19a1a* expression. *Amh* and *cyp19a1a* are genes involved in sex differentiation across all vertebrate taxa [35, 61], and have been reported as male and female like genes, respectively, in several fish species [62, 63]. In our experiment, *cyp19a1a* showed higher expression in females and *amh* in males at 90 dpf. A threshold expression of *amh* could be controlling sexual fate. If *amh* does not reach the required expression level, primordial germ cells will proliferate and maintain *cyp19a1a* levels while *amh* levels decrease. On the contrary, if *amh* expression reaches a certain threshold, germ cells stop proliferating and *cyp19a1a* expression decreases. Although further data is required, this hypothesis seems to be consistent with the findings in turbot and other fish species, and could be a common mechanism controlling the balance between male and female gonad differentiation in fish.

### Female sex differentiation

Turbot GD is in progress at 105–135 dpf, either towards males or females, and sex was easily identified by *cyp19a1a* expression alone at these developmental stages. Several female up-regulated genes were detected during this period of GD (*foxl2*, *vasa*, *tdrd1*, *sox19*, *dnmt1*, *dact1*, *rdh3*), while male gene expression pattern was very similar to undifferentiated fish for the assayed genes, excluding a few classical male-like genes (*amh*, *sox9a*, *sox8*). *Foxl2* is a transcription factor that activates *cyp19a1a* transcription [64] and both genes are strictly co-expressed in mammals [65]. The expression pattern of *Foxl2* was similar to that previously described for *cyp19a1a*, *vasa*, *tdrd1* and *gsdf*. Its expression in turbot is consistent with an activation of *cyp19a1a*; however, the later decrease of *cyp19a1a* while *foxl2* expression is still high in males suggests other roles for *foxl2* not related to *cyp19a1a* activation at early stages of development. *Foxl2* expression has also been described in the male gonad of tilapia (*Oerochromis niloticus*), southern catfish (*Silurus meridionalis*) and goldfish [54, 64, 66]. Also, cells with *cyp19a1a* expression without *foxl2* expression have been reported in medaka [67]. Although the authors did not exclude that those cells had earlier *foxl2* expression, at least *foxl2* did not seem to be essential for *cyp19a1a* expression maintenance [67]. *Foxl2* seems to be involved in the initiation of the female gonad development cascade through the activation of *cyp19a1a* expression in fish, as previously suggested [64], however *foxl2* up-regulation alone is not sufficient for the activation of *cyp19a1a* and it might have additional roles important for gonad development both in males and females.

*Ctnnb1* is another female-like gene with a critical function in female differentiation in mammals, antagonizing *sox9* and blocking testis development, thus promoting ovarian development [68]. *Ctnnb1* is the key downstream component of the canonical *wnt* signaling pathway and our results are in agreement with a conserved female function of this pathway, which has also been shown in zebrafish [69] and rainbow trout [70]. However, in turbot, the *wnt* pathway seems to be at least partially independent of *wnt4*. The highest expression of this gene was found at undifferentiated stages, consistent with a role in early gonad development in both sexes also observed in mammals, although with a different function since in mammals it is responsible for the development of Müllerian ducts [71]. *Wnt4* expression dropped at 90 dpf irrespective of sex, and later was found scarcely up-regulated in females. *Wnt4* is a key female gene in mammals which antagonizes *fgf9* and down-regulates *sox9* expression [72]. *Wnt4* not so clear female-pattern during GD in turbot is likely related to the absence of *fgf9* in teleosts [46]. No dimorphic *wnt4* expression has been observed in rainbow trout (*Oncorhynchus mykiss*) [73], zebrafish [69] or the more distant *Rana rugosa* [74]. *Wnt4* does not show a conserved function in female SD throughout evolution, and the results in our study suggest that it is not involved in the expression of *ctnnb1* in the female gonad development and therefore other WNT proteins could be responsible for activating the *wnt* pathway, which seems to have a conserved female prominent function.

Six genes, *dnmt1*, *rdh3*, *sox19*, *dact1*, *ctnnb1* and *sf1* showed a similar increasing expression pattern in females from 105 to 135 dpf. These genes showed high pair-wise correlation values and also the highest negative correlations with *amh* amongst all the assayed genes. Hence, *amh* down-regulation may be required for the activation of genes involved in female gonad development. The coactivation of these genes in female differentiation does not seem to be related to a specific pathway considering their functional diversity, but with the activation of several concomitant pathways at the beginning of ovarian development, and their up-regulation is possibly connected to the down-regulation of *amh*.

### Male-like genes

Besides *amh*, we found two up-regulated genes in males from 105 dpf onwards: *sox9a* and *sox8*. Furthermore, the heatmap and network analyses suggested that *cyp11a* and *fshb* are related to male development as well, but they did not display a dimorphic expression pattern at any stage. *Sox9a* is an essential player in sex differentiation and its male-like nature seems to be rather conserved throughout evolution, but its relevance seems to be variable [75, 76]. In mammals, this gene is directly activated by *sry* and is responsible and sufficient for fating the male gonad [77], also activating *amh* transcription [78]. In birds, *sox9* is co-expressed with *amh* and induced by the SD gene *dmrt1* [79]. However, in medaka, *sox9* is not required for testis development [60]. In turbot, *sox9a* dimorphic expression is found later than that of *amh*, and its expression is more stable along the assayed stages, suggesting a less important role in GD in this species.

### Genes in the main SD region

*Fxr1* is located in the turbot genome very close to Sma-USCE30 [32], the highest sex-associated marker in this species, within the main SD region at LG5 [30], thus representing a potential SD candidate gene. In this experiment, *fxr1* is highly expressed before the first GD signs and precedes the expression of *cyp19a1a* and *amh*, although at this time its expression is not sexually dimorphic. *Fxr1* is a RNA-binding protein and is an autosomal paralogue of *fmrp* (fragil X mental retardation 1), important for normal female reproductive function and cognition development in humans. Further, *fxr1* It has been related to female gametogenesis in pigs [80] and *Xenopus laevis* [81]. In turbot, *fxr1* position in the functional network, connecting male and female clusters, and its higher expression at low temperatures, suggest that further analysis on this gene in younger individuals looking for a putative dimorphic pattern between sexes or other type of dimorphic cue are desirable. On the contrary *sox2*, also located in the main SD region of turbot [82], did not present a dimorphic expression pattern and also showed a relatively steady expression along all the assayed stages, suggesting no role of this gene in turbot SD.

### Temperature effects

A higher proportion of phenotypic females were obtained at 15 and 18 °C, however sex ratio differences between temperatures were not significant. This effect was not due to genetic segregation distortion, since genotyping of the SD marker strongly suggested that some genetic males developed as females. Although proper male-to-female sex reversal at low temperatures has not been described in fish [83], it has been described in reptiles, with genetic males developing as females [84, 85]. The influence of temperature on sex ratio in turbot was previously reported [31], but it was family-dependent and not always in the same way: in this study, two families presented a higher proportion of females than expected at 23 °C while another family presented more females at 15 °C. In any case, if temperature effects exist in turbot, they seem to be limited and family dependent, however further work should be done to evaluate this issue. Another possible explanation for genetic males developing as phenotypic females is the existence of a family-specific genetic interaction between a secondary SD QTL and the main SD QTL in LG5, which could be responsible for genetic males developing as phenotypic females. Independently of the mechanism, families with higher proportions of females could be very interesting for aquaculture, since it would lead to higher growth rates given the important sex size dimorphism of turbot.

Nevertheless, temperature effects on gene expression are very important to explain possible sex ratio shifts and other effects on sex differentiation. We found temperature effects on the expression of some of the genes assayed: *amh*, *ctnnb1*, *cyp11a*, *dmrt2*, *piwi2*, *sf1*, *sox2*, *sox6*, *sox9a* and *sox17*. Among these, only *sox2* temperature effects were sex independent. This gene showed higher expression both at 15 and 23 °C, which may indicate some kind of stress response. *Sox2* is a transcription factor regulating several genes and it is also involved in the maintenance of stem-cell identity [86].

Among the genes which presented sex dependent temperature effects, *ctnnb1*, *piwi2*, *sf1* and *sox6* showed higher expression at low temperatures in males. Among these, *ctnnb1* is remarkable because it not only occupies a downstream position in the *wnt* pathway [87], which needs to be up-regulated for the development of an ovary in zebrafish [68], but also because it showed temperature-dependent expression differences in our study at 90 dpf, the onset of SD. Genes found upstream in the *wnt* signalling pathway are likely regulated by temperature and responsible for this increase in *ctnnb1* expression in turbot. Consistently with our results, elevated *ctnnb1* expression has been reported connected to low temperatures in rats [88] and tilapia [89], albeit in other tissues. A recent study in oyster found a biased sex ratio towards females related to higher *ctnnb1* expression at lower rearing temperatures [90]. Thus, *ctnnb1* and the wnt pathway are good candidates for future studies aimed at investigating temperature effects on sex ratios in turbot and possibly other fish.

Other two genes showed the opposite pattern in females, with higher expression at higher temperatures: *dmrt2* and *sox17. Dmrt2* was upregulated during gonad development and also expressed in germ cells in the swamp eel (*Monopterus albus*) [91], while in *Rana rugosa* it was expressed in the developing gonad during SD without any dimorphic pattern, suggesting a function both in testicular and ovarian differentiation [92]. S*ox17* has been associated with ovarian development in *Dicentrarchus labrax* [93], although it did not present dimorphic expression in turbot and so, apparently, it is not related to female differentiation in this species. Yet, this gene seemed to have a peak of expression at 90 dpf in both sexes, so it could have some function in early gonad development in turbot for both males and females. In the swamp eel, *sox17* was also expressed both in testis and ovary [94].

Temperature effects are gene and sex specific. Given the labile nature of SD in fish related to specific morphogenetic thresholds, several genes could be responsible for sex ratio shifts. As seen in this study, several genes involved in sex show expression differences due to temperature, and so these genes are potential candidates for sex ratio alterations.

## Conclusions

Turbot sex differentiation is ongoing at 90 dpf and sex can be distinguished by the expression levels of three genes when fish are 5–6 cm length: *cyp19a1a*, *amh* and *vasa*; while later females are easily discriminated by the expression of *cyp19a1a*. The first molecular signs of sex differentiation are the dimorphic expression of these three genes and an increase in the expression of *vasa*, *gsdf* and *tdrd1*, connected with primordial germ cells, suggesting their proliferation from 75 to 90 dpf and an important role in sex differentiation. The primary sex determining gene of turbot remains unknown, since neither *sox2* nor *fxr1*, genes located in the main SD region of turbot, showed expression patterns clearly consistent with this role. Our data suggest that female development has more complex machinery and is strongly regulated, suggesting the involvement of both methylation and splicing mechanisms. Furthermore, we have observed that temperature affects the expression of several genes and suggest that the *Wnt*/*β-catenin* pathway could be a likely candidate to explain possible temperature-induced sex ratio shifts.

The present study, analyzing the expression pattern of many genes related to sex differentiation, has revealed that turbot sex differentiation is a complex process with many factors involved. These results are more compatible with a view of sex determination as a network where the activation or repression of several genes can affect gonad fate. This view of sex determination as a threshold character could help us to understand temperature effects during sex differentiation.

## Methods

### Rearing conditions and sampling

Turbot fertilized eggs were obtained at the IEO (Instituto Oceanográfico de Vigo, Spain) from a stock of wild spawners obtained at Celeiro (Galicia, North-West of Spain) and adapted to captivity. Turbot eggs were incubated in a 140 L cylindrical-conical tank at a temperature of 13–14 °C until hatching (5 days post fertilization, dpf). Hatched larvae were transferred to a 6 fiberglass cylindrical tanks (500 L) with an initial density of 30 larvae/L. The tanks were divided in three groups (2 tanks/group) and temperature was gradually adjusted to 15 °C (cold group), 18 °C (ambient group) and 23 °C (warm group). Water temperature was monitored 6–8 times per day. After metamorphosis the fish were transferred to 6 flat-bottom tanks and the temperatures maintained until the end of the experiment. Gonad samples were taken at 60, 75, 90, 105, 120 and 135 dpf. This period was chosen based on a preliminary analysis on the expression GD key genes (*cyp19a1a*, *amh*, *sox9a*, *vasa*, *foxl2*) every five days from 5 dpf, which did not show any expression changes before 90 dpf. We started our sampling 30 days earlier trying not to miss the expression of a possible sex determining gene. Ten fish per temperature and age were sampled and gonads dissected as accurately as possible considering the size of the fish. A total of 180 samples were used in this study: 6 ages × 3 temperatures/age × 10 fish for each age-temperature combination. In fish of 105 dpf and older, gonads were hemi dissected, one used for quantitative PCR (qPCR) and the other one for histological sexing. Animals were treated according to the Directive 2010/63/UE of the European Parliament and of the Council of 22 September 2010 on the protection of animals used for experimentation and other scientific purposes. All experimental protocols were approved by the Institutional Animal Care and Use Committee of the University of Santiago de Compostela (Spain).

### Sexing by histology and molecular markers

Samples for histological analysis were kept in 4 % paraformaldehyde buffer overnight, rinsed with PBS the next day and stored in 70 % ethanol until further analysis. Samples were dehydrated and embedded in paraffin, cut at 7 μm thick and stained with hematoxylin-eosin for the determination of phenotypic sex. Additionally, all samples were genetically sexed using the SmaUSC-E30 marker, which demonstrated a ~98 % accuracy for the identification of genetic sex in turbot [30]. To establish the association between sex and alleles at this marker, parents and grandparents of each family were genotyped, and the expected genotypes of male and female offspring obtained following [32].

### RNA isolation and cDNA synthesis

Upon dissection, samples for qPCR were immediately embedded in RNAlater for preservation (Qiagen, Valencia, CA). Total RNA was extracted by homogenization in TRIzol (Invitrogen, Paisley, UK) following the manufacturer’s protocol. Extracted RNA was treated with RNase-free Recombinant *DNaseI* (Roche Diagnostics, Mannheim, DE) and RNA concentration was assessed by spectrophotometry and its quality checked using an Agilent 2100 bionalyzer (Agilent Technologies, Santa Clara, US). RNA (1.2 μg) was reverse transcribed by random primers using Affinity Script Multiple Temperature cDNA Synthesis Kit (Agilent Technologies) following the manufacturer’s protocol and then diluted 1:2 with nuclease-free water.

### Quantitative PCR

The 29 target genes were selected by: i) their importance for GD in other fish species; ii) previous data from our group in turbot [82, 95]; and iii) previous results from Ribas et al. (submitted) (Additional file 2). The 29 target genes are: *amh*, *ar1*, *ctnnb1*, *cyp11a*, *cyp19a1a*, *dact1*, *dmrt2*, *dnmt1*, *fig-h*, *foxl2*, *fshb*, *fxr1*, *gsdf*, *hh1*, *hsp27*, *lhx8*, *piwi2*, *ptges3*, *rdh3*, *sf1*, *sox2*, *sox6*, *sox8*, *sox9a*, *sox17*, *sox19*, *tdrd1*, *vasa*, *wnt4* and *zar1.* (GenBank NCBI database accession numbers available in Additional file 6).

qPCR was performed on a Stratagene Mx3005P thermocycler (Agilent Technologies) using Brilliant III Ultra-Fast SYBR Green QPCR Master Mix in a final volume of 12.5 μL following the manufacturer’s protocol with 1 μL of cDNA per reaction. Specific primers for targeted genes were designed using Primer3 [96] from sequences obtained from the turbot EST database enriched with sex differentiation-related organs (gonad and brain [97]). When possible, primers were designed spanning different exons (Additional file 6). Primer concentration was 300 nM and each sample was run in duplicate. The cycling parameters were: 50 °C for 2 min, 95 °C for 10 min, followed by 40 cycles of amplification at 95 °C for 15 s and 60 °C for 1 min. After amplification, a dissociation step was performed raising the temperature from 65 to 95 °C to create a melting curve and ensure the presence of a single amplification product. Specificity for each primer pair was also confirmed by PCR product sequencing. In every PCR plate, non-template controls were included to confirm the absence of contamination. In addition, the same three samples were run in triplicate in every plate in order to correct inter-assay variation. qPCR data were obtained by the MxPro software (Agilent Technologies) and quantification cycle values (Cq) calculated for each replicate and then averaged to obtain the final Cq value. Three reference genes (*ubq, rps4, rpl17*) were used for normalization and LinRegPCR software [98] was used for efficiency determination following the recommendations in [99]. qPCR was performed in all the 180 samples for every gene. Samples with missing Cq values or inconsistencies between replicates (Cq difference > 1 cycle) were removed. Raw Cq values were transformed to the final fold difference values (FD) following the equations present in [100]. Briefly, Cq values were normalized using the reference genes, efficiency corrected, log transformed and finally mean centered to obtain mean centered fold change values which were used for statistical analysis. All expression data is in Additional file 7, presented together with all the data collected for every sample.

### Statistical analysis

Statistical analyses were performed using R (version 3.0.2) [101]. Pearson correlations for the heatmap were obtained using the “cor” function. Principal component analysis (PCA) was computed by the “prcomp” function. Length and gene expression differences between sexes and ages were checked by Mann–Whitney tests (*P* < 0.05) since our data mostly did not conform to a normal distribution. Discriminant analysis was performed using the “lda” function on the “MASS” package [102]. A Chi-square test was performed to assess if sex ratio difference between temperatures were significative. Multiple regression (*p* < 0.05) was used to assess temperature effects on gene expression, introducing temperature and length in the model. The gvlma function of the gvlma R package was used to check if our dataset met the assumptions of the multiple regression. Furthermore, we performed two additional tests for every temperature significant effect on gene expression: i) a moderation analysis, to check if length was modulated by the temperature, a temperature-length interaction term was added to our model checking if the new model improved the previous one; and ii) a mediation analysis by Sobel test, to explore if the detected temperature effect on gene expression is partially or fully explained by size differences between individuals.

### Co-localization of targeted genes with sex-related QTLs

Several SD related QTLs were previously reported in turbot [30], and therefore, we considered relevant to establish the mapping position of the targeted genes regarding these QTLs in the last turbot map [29]. For this, we established the correspondence between the turbot linkage groups and the scaffolds of the recently sequenced turbot genome (Figueras et al., unpublished) using the mapped markers and their sequences. Target gene sequences were located in the turbot genome using local blast [103] and then placed in the linkage map using the correspondence between linkage groups and scaffolds as far as accurately depending on the availability of markers in the vicinity.

### Weighted correlation network analysis

Weighted correlation network analysis was performed in R (version 3.0.2) [101] using the WGCNA package [104] following the author’s tutorial. Co-expression networks were built for our genes and Cytoscape 3.0.2 was used to visualize the network [105]. This allowed us to obtain information about the functional relationships between the target genes.

## Availability of supporting data

The data set supporting the results of this article (expression values for every gene in each sample and the data collected for every sample) is included in the Additional file 7.

## Additional files

Additional file 1:
**Male and female length by age.** Mean length (centimeters) by age (days post fertilization) is shown in a boxplot for males and females separately. Females are represented in magenta and males in blue. (PNG 6 kb) Additional file 2:
**Brief information on the genes studied by qPCR.** A brief description on why each assayed gene was chosen for this study is presented along with supporting references. (DOCX 28 kb) Additional file 3:
**Genes without sex dimorphic expression.** Fold change values for those genes without significant differences between males and females at any age. Fold change values for each sample were plotted according to both its length, in cm, and its age, in days post fertilization. Female samples are shown in magenta and male samples in blue. In the FC/length figure for each gene non-linear trend lines were calculated by loess regression. In the FC/age figure, error bars represent the standard error of the mean. (PNG 138 kb) Additional file 4:
**Genes showing temperature differences also influenced by growth.** Mean fold change gene expression values at 15, 18 and 23 °C in the whole dataset are shown for males (light blue background) and females (pink background). Error bars represent standard deviation. Significant differences between temperatures are indicated by *(0.01 < *p* < 0.05), **(0.001 < *p* < 0.01), ***(0.0001 < *p* < 0.001) or ****(*p* < 0.0001). Red asterisks indicate that fish length has an influence on the temperature differences. (PNG 956 kb) Additional file 5:
**Temperature effects on** ***ctnnb1*** **expression at 90 dpf.** Mean fold change gene expression values at 15, 18 and 23 °C at 90 dpf are shown for ctnnb1. Error bars represent standard deviation. Significant differences between temperatures are indicated by *(0.01 < *p* < 0.05) or **(0.001 < *p* < 0.01). (TIFF 478 kb) Additional file 6:
**PCR primers.** Forward and reverse primers for each amplified gene are shown. Furthermore, GenBank accession numbers for template sequences for primer design and amplicon size are shown. (DOCX 16 kb) Additional file 7:
**Complete dataset.** Expression values for every gene for each sample, together with sample age, length, weight, rearing temperature, genetic sex and histological sex. (XLSX 82 kb)
