# Supplementary figures and images for: Gene expression analysis at the onset of sex differentiation in turbot (Scophthalmus maximus)
Source: BMC Genomics. 2015 Nov 18;16:973. doi: 10.1186/s12864-015-2142-8 (PMC4652359; doi:10.1186/s12864-015-2142-8)

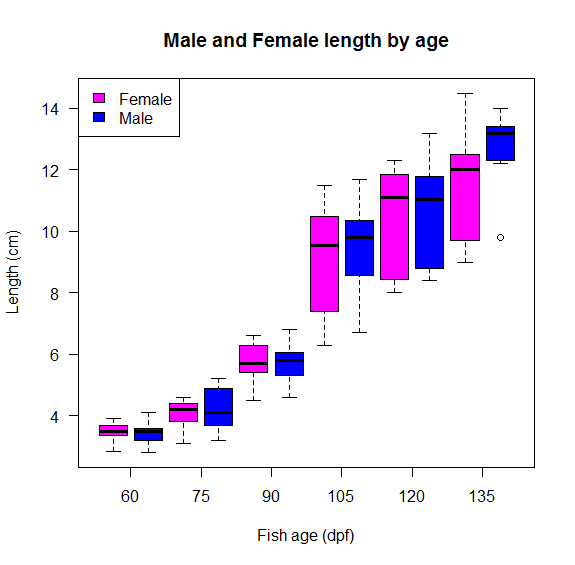

Supplement: Additional file 1: — Male and female length by age. Mean length (centimeters) by age (days post fertilization) is shown in a boxplot for males and females separately. Females are represented in magenta and males in blue. (PNG 6 kb) [file 12864_2015_2142_MOESM1_ESM.png]

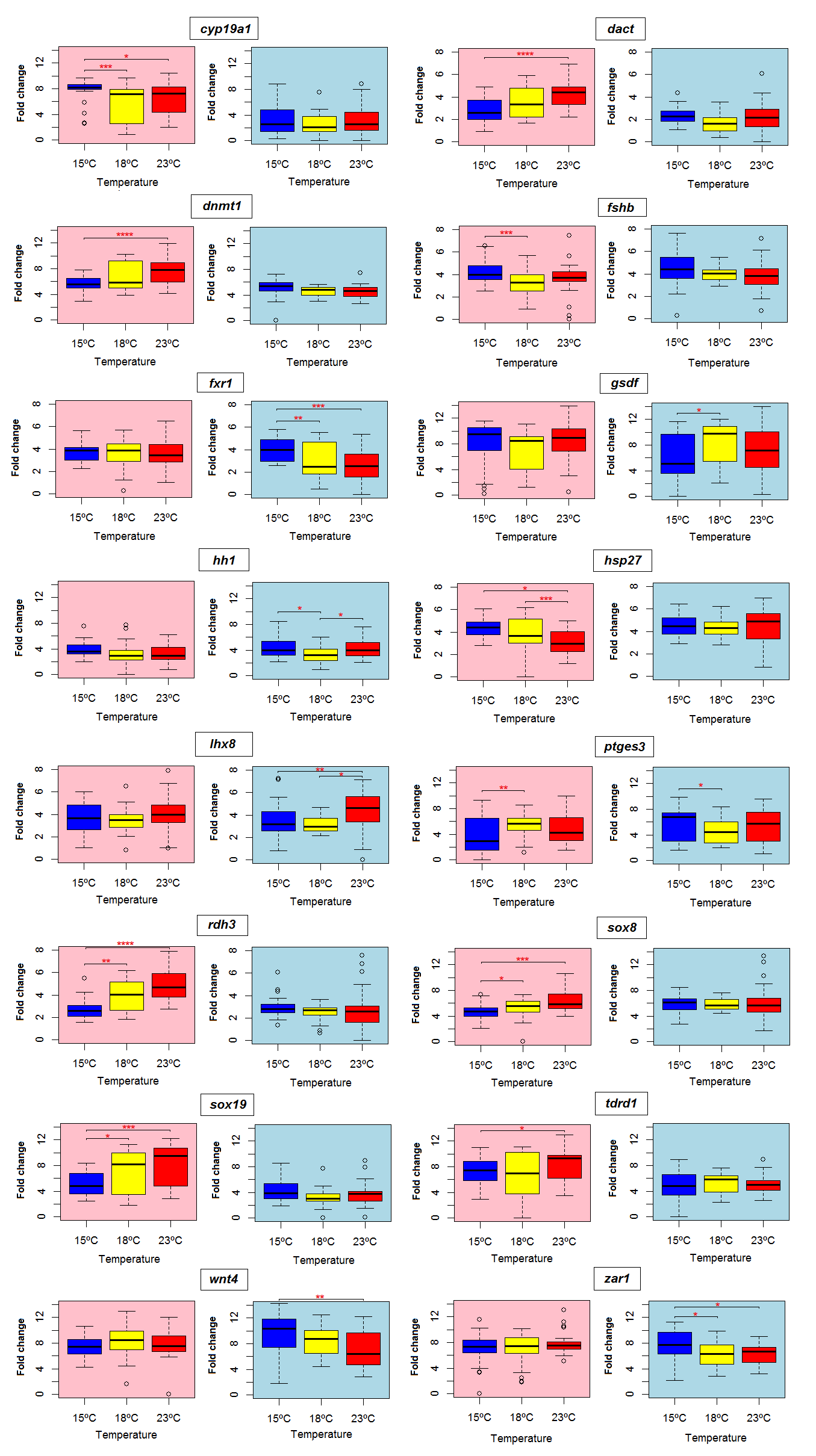

Supplement: Additional file 3: — Genes without sex dimorphic expression. Fold change values for those genes without significant differences between males and females at any age. Fold change values for each sample were plotted according to both its length, in cm, and its age, in days post fertilization. Female samples are shown in magenta and male samples in blue. In the FC/length figure for each gene non-linear trend lines were calculated by loess regression. In the FC/age figure, error bars represent the standard error of the mean. (PNG 138 kb) [file 12864_2015_2142_MOESM3_ESM.png]

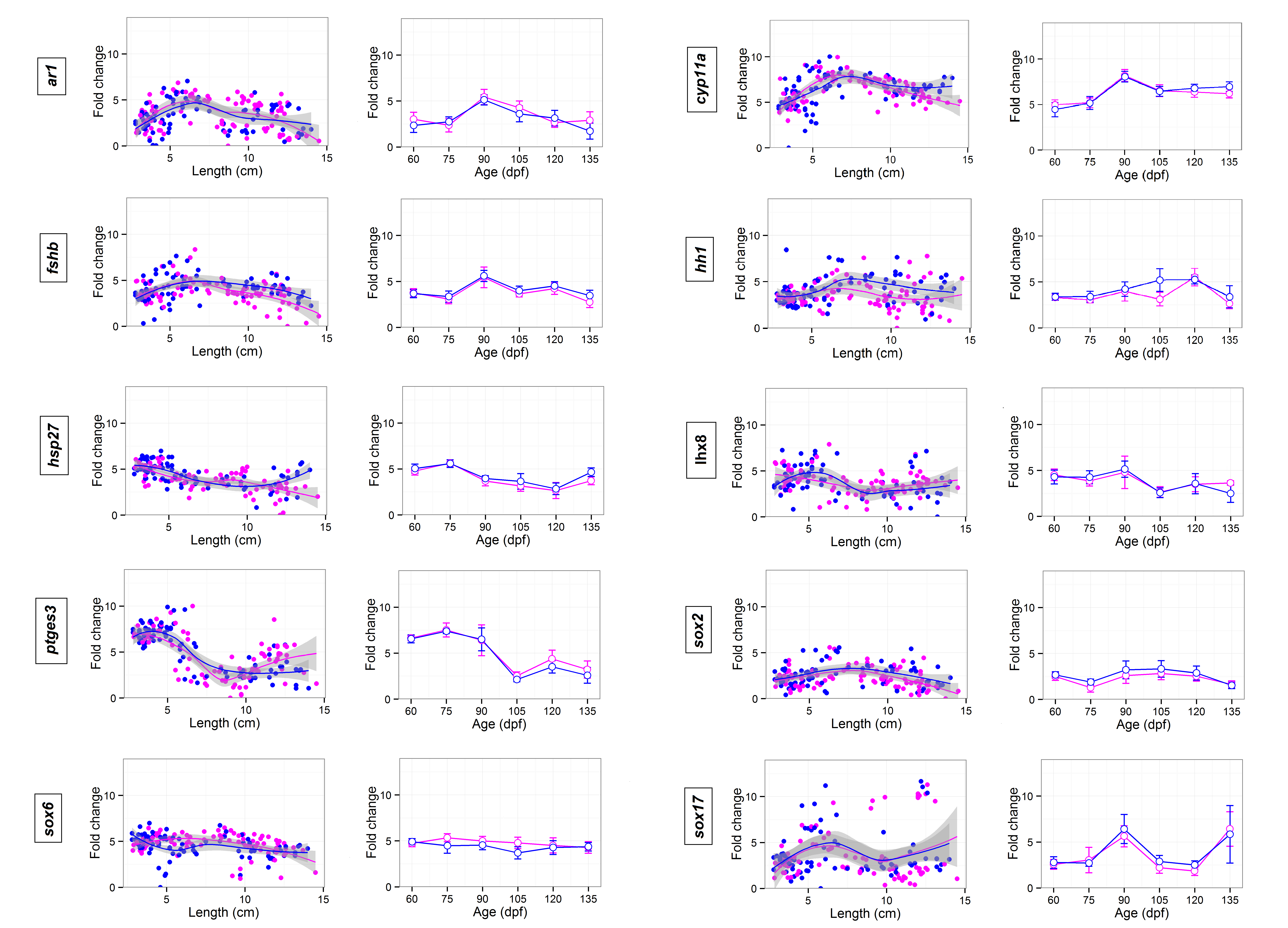

Supplement: Additional file 4: — Genes showing temperature differences also influenced by growth. Mean fold change gene expression values at 15, 18 and 23 °C in the whole dataset are shown for males (light blue background) and females (pink background). Error bars represent standard deviation. Significant differences between temperatures are indicated by *(0.01 < p < 0.05), **(0.001 < p < 0.01), ***(0.0001 < p < 0.001) or ****(p < 0.0001). Red asterisks indicate that fish length has an influence on the temperature differences. (PNG 956 kb) [file 12864_2015_2142_MOESM4_ESM.png]

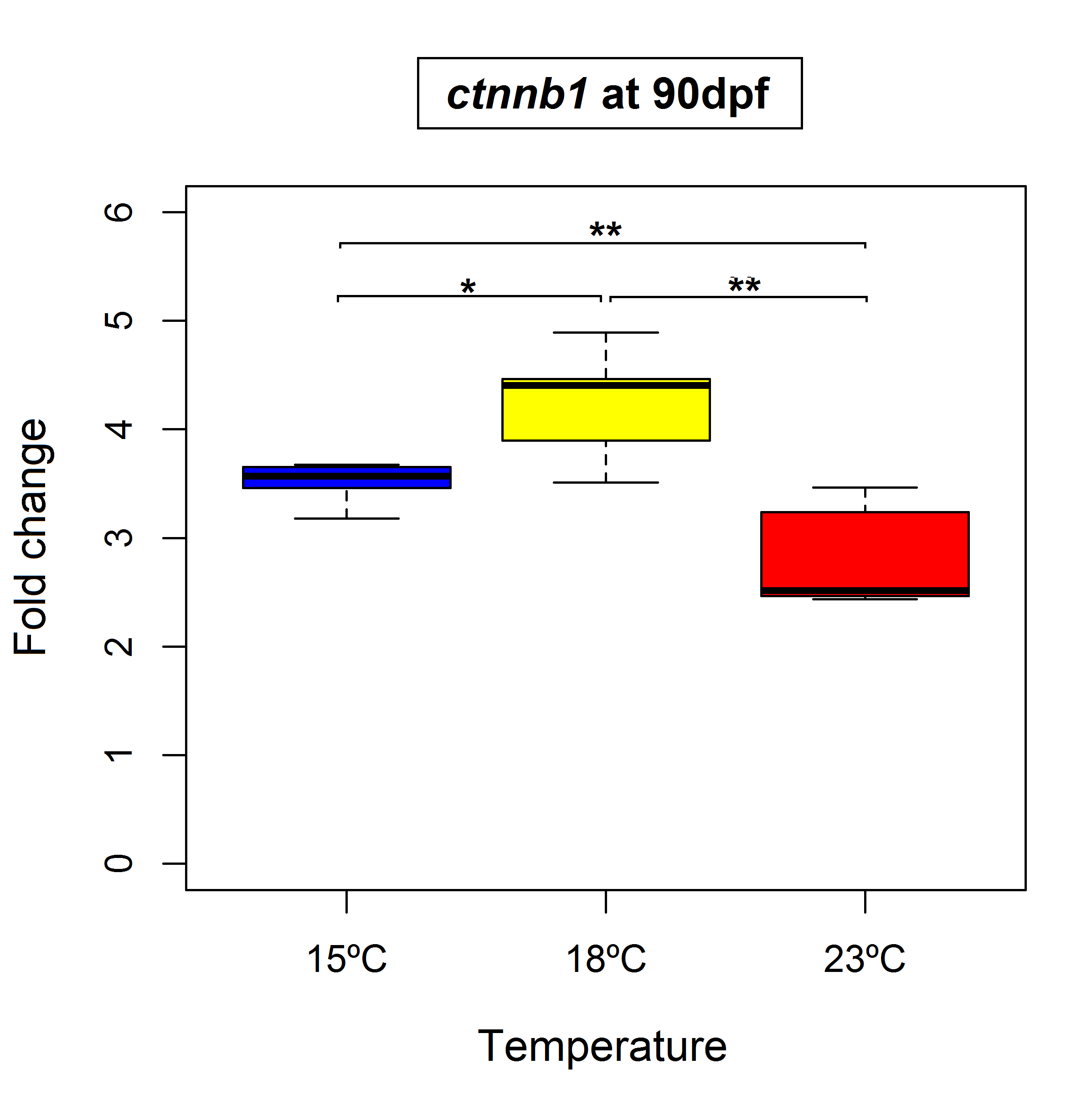

Supplement: Additional file 5: — Temperature effects on ctnnb1 expression at 90 dpf. Mean fold change gene expression values at 15, 18 and 23 °C at 90 dpf are shown for ctnnb1. Error bars represent standard deviation. Significant differences between temperatures are indicated by *(0.01 < p < 0.05) or **(0.001 < p < 0.01). (TIFF 478 kb) [file 12864_2015_2142_MOESM5_ESM.tif]
