# Supplementary material for: Gene expression analysis at the onset of sex differentiation in turbot (Scophthalmus maximus)
Source: BMC Genomics. 2015 Nov 18;16:973. doi: 10.1186/s12864-015-2142-8 (PMC4652359; doi:10.1186/s12864-015-2142-8)
Supplement: Additional file 2: — Brief information on the genes studied by qPCR. A brief description on why each assayed gene was chosen for this study is presented along with supporting references. (DOCX 28 kb) [file 12864_2015_2142_MOESM2_ESM.docx]

Additional file 2. A brief description on why each assayed gene was chosen for this study is presented along with supporting references

| **Gene Symbol** | **Annotation** | **Function/Relevance** |
| --- | --- | --- |
| *amh* | Müllerian-inhibiting factor | Belongs to the TGF (transforming growth factor) super family. Regression of the Müllerian ducts in male mammals (Cate et al. 1986) |
| *ar1* | Androgen receptor 1 | Male differentiation (Walters et al. 2010) |
| *ctnnb1* | catenin β-1 | Downstream effector of the canonical *Wnt* signaling pathway (Kühl and Wedlich, 1997) |
| *cyp11a1* | Cytochrome P450 11A1 | Testosteron biosynthetic process (Strushkevich et al. 2011) |
| *cyp19a1a* | Aromatase | Catalyzes the formation of estrogens from androgens (Corbin et al. 1988), in fish is essential for female development (Piferrer and Guiguen, 2008). |
| *dact1* | Dapper homolog 1 | Function in stabilizing CTNNB1 (catenin β-1). Promotes the membrane localization of CTNNB1 (Sensiate et al. 2014) |
| *dmrt2* | Doublesex- and mab-3-related transcription factor 2 | Expressed in testis. Not required for sex differentiation in mice (Kim et al. 2003) |
| *dnmt1* | DNA (cytosine-5)-methyltransferase 1 | Methylates CpG residues. Preferentially methylates hemimethylated DNA (Pradhan et al. 2008) |
| *foxl2* | Forkhead box protein L2 | Critical factor essential for ovary differentiation and maintenance (Ottolenghi et al. 2005) |
| *fshb* | Follitropin subunit β | Stimulates development of follicle and spermatogenesis in the reproductive organs (Wreford et al. 2001;) |
| *fxr1* | Fragile X mental retardation syndrome-related protein 1 | Located at the main SD turbot QTL (Martinez et al. 2009) |
| *gsdf* | Gonadal soma derived factor | Belongs to the (transforming growth factor) TGF−β super family, gonad specific expression (Gautier et al. 2011), in medaka has been proposed as the sex determining gene (Myosho et al. 2012) |
| *hh1* | Histone H1 | Chromatin structure protein (Th’ng et al. 2005) |
| *hsp27* | Heat shock 27 kDa protein | Regulates androgen receptor levels (Stope et al. 2012) |
| *lhx8* | Lim homeobox 8 | Involved in ovarian formation and folliculogenesis in mouse (Choi et al. 2008; Jagarlamudi and Rajkovic 2012) |
| *piwi2* | Piwi-like protein 2 | Germ cell differentiation. Essential for the germline integrity, repressing transposable elements (Aravin et al. 2007) |
| *ptges3* | Prostaglandin E synthase 2 | Prostaglandin biosynthesis (Murakami et al. 2003) |
| *rdh3* | Retinol dehydrogenase 3 | Acts on retinol bound on cellular retinol-binding protein (CRBP) (Chai et al. 1995). Retinoic acid is involved in germ cell meiosis entry (Bowles and Koopman, 2010) |
| *sf1* | S factor 1 | Necessary for spliceosome assembly (Wang et al. 1999) |
| *sox2* | Transcription factor *SOX-2* | Negative regulation of canonical Wnt signaling pathway (Mansukhani et al. 2005) |
| *sox6* | Transcription factor SOX-6 | Cellular response to transforming growth factor beta stimulus (Kim and Im, 2011) |
| *sox8* | Transcription factor SOX-8 | Involved in male SD, reinforcement of *sox9* action (Barrionuevo et al. 2009) |
| *sox9a* | Transcription factor SOX-9 | Male sex differentiation (Cameron and Sinclair, 1997) |
| *sox17* | Transcription factor SOX-17 | Involved in spermatogenesis (Wang et al., 2005) also is related to ovarian development (Navarro-Martín et al., 2009). Inhibits Wnt signaling, promotes degradation of activated CTNNB1 (Liu et al. 2010) |
| *sox19* | Transcription factor *Sox-19* | Transcriptional activator belonging to the sox B1 group (Okuda et al. 2006), involved in ovarian differentiation (Navarro-Martín et al., 2012) |
| *tdrd1* | Tudor domain-containing protein 1 | Participating in the repression transposable elements preventing their mobilization in humans has a central role in spermatogenesis (Reuter et al. 2009) |
| *vasa* | Probable ATP-dependent RNA helicase DDX4 | Germ cell marker (Castrillon et al. 2000) |
| *wnt4* | Protein Wnt-4 | Gonad development and female SD (Jordan et al. 2001). |
| *zar1* | Zygote arrest protein 1 | Essential for female fertility (Wu et al. 2003) |

- Aravin AA, Sachidanandam R, Girard A, Fejes-Toth K, Hannon GJ. Developmentally regulated piRNA clusters implicate MILI in transposon control. Science. 2007;316:744-747.
- Barrionuevo F, Georg I, Scherthan H, Lecureuil C, Guillou F, Wegner M, et al. Testis cord differentiation after the sex determination stage is independent of Sox9 but fails in the combined absence of Sox9 and Sox8. Dev Biol. 2009;327:301-312.
- Bowles J, Koopman P. Sex determination in mammalian germ cells: extrinsic versus intrinsic factors. Reproduction. 2010;139(6):943-958.
- Cameron FJ, Sinclair AH. Mutations in SRY and SOX9: testis-determining genes. Hum Mutat. 1997;9:388-395.
- Castrillon DH, Quade BJ, Wang TY, Quigley C, Crum CP. The human VASA gene is specifically expressed in the germ cell lineage. Proc Natl Acad Sci USA. 2000;97:9585-9590.
- Cate RL, Mattaliano RJ, Hession C, Tizard R, Farber NM, Cheung A, et al. Isolation of the bovine and human genes for Müllerian inhibiting substance and expression of the human gene in animal cells. Cell. 1986;45:685-698.
- Chai X, Boerman MHEM, Zhai Y, Napoli JL. Cloning of a cDNA for liver microsomal retinol dehydrogenase. A tissue-specific, short-chain alcohol dehydrogenase. J Biol Chem. 1995270:3900-3904.
- Choi Y, Ballow DJ, Xin Y, Rajkovic A. Lim homeobox gene, Lhx8, is essential for mouse oocyte differentiation and survival. Biology of Reproduction. 2008;79(3):442-449.
- Corbin CJ, Graham-Lorence S, McPhaul M, Mason JI, Mendelson CR, Simpson ER. Isolation of a full-length cDNA insert encoding human aromatase system cytochrome P-450 and its expression in nonsteroidogenic cells. Proc Natl Aca Sci USA. 1988;85:8948-8952.
- Gautier A, Sohm F, Joly JS, Le Gac F, Lareyre JJ. The proximal promoter region of the zebrafish gsdf gene is sufficient to mimic the spatio-temporal expression pattern of the endogenous gene in Sertoli and granulose cells. Biol Reprod. 2011;85:1240-1251.
- Jagarlamudi K, Rajkovic A. Oogenesis: Transcriptional regulators and mouse models. Molecular and Cellular Endocrinology. 2012;356(1-2):31-39.
- Jordan BK, Mohammed M, Ching ST, Delot E, Chen XN, Dewing P, et al. Up-regulation of wnt-4 signaling and dosage-sensitive sex reversal in humans. Am J Hum Genet. 2001;68:1102-1109.
- Kim HJ, Im GI. Electroporation-mediated transfer of SOX trio genes (SOX-5, SOX-6, and SOX-9) to enhance the chondrogenesis of mesenchymal stem cells. Stem Cells Dev. 2011;20:2103-2114.
- Kim S, Kettlewell JR, Anderson RC, Bardwell VJ, Zarkower D. Sexually dimorphic expression of multiple doublesex-related genes in the embryonic mouse gonad. Gene Expr Patterns. 2003;3:77-82.
- Kühl M, Wedlich D. Wnt signaling goes nuclear. Bioessays. 1997;19:101-104.
- Kumar TR, Wang I, Lu N, Matzuk MM. Follicle stimulating hormone is required for ovarian follicle maturation but not male fertility. Nat Genet. 1997;15:201-204.
- Liu X, Luo M, Xie W, Wells JM, Goodheart MJ, Engelhardt JF. Sox17 modulates Wnt3A/beta-catenin-mediated transcriptional activation of the Lef-1 promoter. Am J Physiol. 2010;299:694-710.
- Mansukhani A, Ambrosetti D, Holmes G, Cornivelli L, Basilico C. Sox2 induction by FGF and FGFR2 activating mutations inhibits Wnt signaling and osteoblast differentiation. J Cell Biol. 2005;168:1065-1076.
- Martinez P, Bouza C, Hermida M, Fernández J, Toro MA, Vera M, et al. Identification of the major sex-determining región of turbot (Scophthalmus maximus). Genetics. 2009;183:1443-1452.
- Murakami M, Nakashima K, Kamei D, Masuda S, Ishikawa Y, Ishii T, et al. Cellular prostaglandin E2 production by membrane-bound prostaglandin E synthase-2 via both cyclooxygenases-1 and -2. J Biol Chem. 2003;278:37937-37947.
- Myosho T, Otake H, Masuyama H, Matsuda M, Kuroki Y, Fujiyama A, et al. Tracing the emergence of a novel sex-determining gene in medaka, Oryzias luzonensis. Genetics. 2012;191: 163-170.
- Navarro-Martín L, Galay-Burgos M, Piferrer F, Sweeney G. Characterisation and expression during sex differentiation of Sox19 from the sea bass Dicentrarchus labrax. Comp Biochem Physiol. 2012;163:316-323.
- Navarro-Martín L, Galay-Burgos M, Sweeney G, Piferrer F. Different sox17 transcripts during sex differentiation in sea bass, Dicentrarchus labrax. Mol Cell Endocrinol. 2009;299:240-251.
- Okuda Y, Yoda H, Uchikawa M, Furutani-Seiki M, Takeda H, Kondoh H, et al. Comparative genomic and expression analysis of group B1 sox genes in zebrafish indicates their diversification during vertebrate evolution. Dev Dyn. 2005;235:811-825.
- Ottolenghi C, Omari S, Garcia-Ortiz JE, Uda M, Crisponi L, Forabosco A, et al. Foxl2 is required for commitment to ovary differentiation. Human Molecular genetics. 2005;14:2053-2062.
- Piferrer F, Guiguen Y. Fish gonadogenesis. Part II: Molecular biology and genomics of sex differentiation. Reviews in Fisheries Science. 2008;16:35-55.
- Pradhan M, Esteve PO, Chin HG, Samaranayke M, Kim GD, Pradhan S. CXXC domain of human DNMT1 is essential for enzymatic activity. Biochemistry. 2008;47:10000-10009.
- Reuter M, Chuma S, Tanaka T, Franz T, Stark A, Pillai RS. Loss of the Mili-interacting Tudor domain-containing protein-1 activates transposons and alters the Mili-associated small RNA profile. Nat Struct Mol Biol. 2009;16:639-646.
- Sensiate LA, Sobreira DR, Da Veiga FC, Peterlini DJ, Pedrosa AV, Rirsch T, et al. Dact gene expression profiles suggest a role for this gene family in integrating Wnt and TGF-β signaling pathways during chicken limb development. Dev Dyn. 2014;243:428-439.
- Stope MB, Schubert T, Staar D, Ronnau C, Streitborger A, Kroeger N, et al. Effect of the heat shock protein HSP27 on androgen receptor expression and function in prostate cancer cells. World J Urol. 2012;30:327-331.
- Strushkevich N, MacKenzie F, Cherkesova T, Grabovec I, Usanov S, Park HW. Structural basis for prenenolone biosynthesis by the mitochondrial monooxygenase system. Proc Natl Acad Sci USA. 2011;108:10139-10143.
- Th’ng JP, Sung R, Ye M, Hendzel MJ. H1 family histones in the nucleus. Control of binding and localization by the C-terminal domain. J Biol Chem. 2005;280:27809-27814.
- Walters KA, Simanainen U, Handelsman DJ. Molecular insights into androgen actions in male and female reproductive function from androgen receptor knockout models. Hum Reprod Update. 2010;16:543-558.
- Wang X, Bruderer S, Rafi Z, Xue J, Milburn PJ, Kraemer A, et al. Phosphorylation of splicing factor SF1 on Ser20 by cGMP-dependent protein kinase regulates spliceosome assembly. EMBO J. 1999;18:4549-4559.
- Wreford NG, Kumar TR, Matzuk MM, de Kretser DM. Analysis of the testicular phenotype of the follicle-stimulating hormone beta-subunit knockout and the activing type II receptor knockout mice by stereological analysis. Endocrinology. 2001;142:2916-2920.
- Wu X, Wang P, Brown CA, Zilinski CA, Matzuk MM. Zygote arrest 1 (Zar1) is an evolutionarily conserved gene expressed in vertebrate ovaries. Biol Reprod. 2003;69:861-867.
