# Supplementary material for: Gene expression analysis at the onset of sex differentiation in turbot (Scophthalmus maximus)
Source: BMC Genomics. 2015 Nov 18;16:973. doi: 10.1186/s12864-015-2142-8 (PMC4652359; doi:10.1186/s12864-015-2142-8)
Supplement: Additional file 6: — PCR primers. Forward and reverse primers for each amplified gene are shown. Furthermore, GenBank accession numbers for template sequences for primer design and amplicon size are shown. (DOCX 16 kb) [file 12864_2015_2142_MOESM6_ESM.docx]

Additional file 4. qPCR primers

| Gene | Accession number | Primer F (5’ -> 3’) | Primer R (5’ -> 3’) | Product length (bp) |
| --- | --- | --- | --- | --- |
| *amh* | KR019996 | CCAGGGCGGACCCCGATAAC | TGGCTGTGTTTGGACCCACGAG | 99 |
| *ar1* | KR019997 | TCGGATGCACGTCTCCACCA | GGAGGGAGTCCAGGAGTCGGG | 275 |
| *ctnnb1* | KP701006 | AGCTGTGTCGGTCGCGGTTT | GCCAGCCTCTGGACGTTGGTG | 404 |
| *cyp11a* | KR019998 | TCTGGGTTTGATGCTGGACT | ACCTTGGTTGAAGATCCCGTC | 178 |
| *cyp19a1a* | JQ403643 | CAGCGAGGAAGCTGGCAAACA | ACACGCAGACTCGGCTTTTTACATC | 148 |
| *dact1* | KR019999 | TCAGAGGGCAAAAATGGGCT | ACTTCAGTGGGCTTCCTGTG | 144 |
| *dmrt2* | KP677565 | GACTTTCTGTCCAAGCCCCT | GGGCGTGGGTCTTTTCAGTA | 91 |
| *dnmt1* | KR019994 | GGAGTACGCGCCCATCTTT | GTCCTCCGTGAAGCAGTTGA | 169 |
| *fig-h* | KT005392 | TGGTCTTTGGGAGTGGGGA | TGAATCGAGCAGTGTCCCAAA | 121 |
| *foxl2* | KR020000 | GGCGGGGAGAGGAAGGGGAAT | ATCCGGCGGCGTCTCCTGTA | 89 |
| *fshb* | KP658394 | TGCAAACTGGCCAACATCAC | CCGTTAATGTGCTTCGCCTC | 179 |
| *fxr1* | KR020001 | AGGTGCCCTTCAGTGATGTC | TCTCGTTGTAGGTGGCATCA | 200 |
| *gsdf* | KP677566 | CTGGGCTGGAACAACTGGAT | GGCACCATTTCCTGGGAGTT | 173 |
| *hh1* | KR020002 | AGAGAGCCAAGTATCGGAGG | ATCCTTCAGCCTTCAGAGCC | 132 |
| *hsp27* | KR020003 | AGGAGAGGAAGGATGAGCACGGC | TTGTTGTCGGCGGTGACGGG | 191 |
| *piwi2* | KR020004 | ACAACACAGCGAACCTCACA | GGCATACTTGCATGGTGCTG | 113 |
| *ptges3* | KP677567 | TCTACGACCGCACCATCAAC | TCATGCTCCCAGTCTCTCCA | 133 |
| *rdh3* | KP658395 | CTGACGACCACACACCTTGA | GCGACTCCAGCATTGTTCAC | 119 |
| *rpl17* | DQ848879 | ACCAGTGCGTCCCCTTCA | CTCATCTTCGGAGCCTTGTTC | 214 |
| *rps4* | FE943956 | CAACATCTTCGTCATCGGCAAGG | ATTGAACCAGCCTCAGTGTTTAGC | 143 |
| *sf1* | KR020005 | TCACCAACACCACCCTCTGT | CCATGAGGGACAGGTACTCC | 155 |
| *sox2* | KR020006 | ACAGACAAATGTCGGGGTTGGGGA | CCGGCTCCTCTCGAGCTTCCT | 147 |
| *sox6* | JQ403637 | CCCATTTCTCCCTCCTCTCT | CCTTTCCGAGGAGACTGTTG | 193 |
| *sox8* | KR020007 | AAGACGCTGGGGAAACTGT | CGGGGCTGGTACTTGTAGTC | 138 |
| *sox9a* | JQ300535 | ATCAGTACCCACACCTGCATAAC | TCAGCCTCCTCCACGAACG | 103 |
| *sox17* | JQ403638 | TGTTCGGGAAGCAGGTGAAAGGT | CTTGTTGCCATTTTAGGGGACAGT | 92 |
| *sox19* | JQ403639 | ACCGAGCGGTTTGTGCCTTG | TCCTCTGGATGCAGTGCTGATTGT | 122 |
| *lhx8* | KP896516 | TTCACCAGCGTTCATTCGTC | CACCGAACTACACAAGCAGA | 280 |
| *tdrd1* | KP896515 | TGAGCCTTTGGTGTGGTCTT | ACCATAGCCCGATACCATGC | 254 |
| *ubq* | FE946708 | GCGTGGTGGCATCATTGAGC | CTTCTTCTTGCGGCAGTTGACAG | 124 |
| *vasa* | JX235364 | CTTAGCTGTGGGCGTGGTGGG | ACGTTCTCCTGGCACATCAACG | 190 |
| *wnt4* | KR020008 | TTGGCAAGGTGGTCACGCAGG | AACGCCACACTGGCTGCTGAG | 76 |
| *zar1* | KR020009 | ACGCCTTCAACAACCAGCAG | GATGTCCTCCCACGCGATATG | 118 |

Forward and reverse primers for each amplified gene are shown. Furthermore, GenBank accession numbers for template sequences for primer design and amplicon size are shown.
